# Incidence of grass and weed sensitization in Bangkok, Thailand: a clinical study

**DOI:** 10.3389/fpubh.2024.1301095

**Published:** 2024-03-28

**Authors:** Sirirat Aud-in, Yotin Juprasong, Bannapuch Pinkaew, Kanokporn Talek, Pongsakorn Tantilipikorn, Wisuwat Songnuan

**Affiliations:** ^1^Department of Plant Science, Faculty of Science, Mahidol University, Bangkok, Thailand; ^2^Systems Biology of Diseases Research Unit, Faculty of Science, Mahidol University, Bangkok, Thailand; ^3^Graduate Program in Toxicology, Faculty of Science, Mahidol University, Bangkok, Thailand; ^4^Center of Excellence on Environmental Health and Toxicology (EHT), Office of the Permanent Secretary (OPS), Ministry of Higher Education, Science, Research and Innovation (MHESI), Bangkok, Thailand; ^5^Department of Biochemistry, Faculty of Medicine, Srinakharinwirot University, Bangkok, Thailand; ^6^Department of Otorhinolaryngology, Division of Rhinology and Allergy, Faculty of Medicine Siriraj Hospital, Mahidol University, Bangkok, Thailand; ^7^Center of Research Excellence in Allergy and Immunology, Faculty of Medicine Siriraj Hospital, Mahidol University, Bangkok, Thailand

**Keywords:** allergic rhinitis, airborne pollen, co-sensitization, pollen allergy, Southeast Asia, public health

## Abstract

**Background:**

Allergic rhinitis (AR) is a prevalent public health concern globally, significantly impacting quality of life. In Thailand, the prevalence of AR is rising, with grass and weed pollen identified as primary outdoor triggers.

**Objectives:**

This study aimed to (1) assess patterns of pollen sensitization in Thai AR patients and (2) investigate correlations between demographics/clinical data and SPT results.

**Methods:**

A total of 121 individuals aged ≥18 years with clinically diagnosed AR were recruited. Skin prick testing (SPT) was performed using a panel of commonly encountered tropical grass and weed pollen extracts. SPT wheal sizes and clinical symptom scores were recorded. Correlations between SPT outcomes and symptom scores were analyzed.

**Results:**

Among the participants, 104 (85.95%) exhibited positive SPT reactions to at least one pollen type. Nutsedge (76/121), para grass (57/121), and Bermuda grass (48/121) were the most frequently identified allergens. Hurricane grass elicited the strongest reaction, evidenced by the highest average wheal size (6.2 mm). Poly-sensitization was observed in 77 (63.6%) of the SPT-positive individuals, with most cases involving two different pollen extracts (35/77). Notably, AR severity positively correlated with both average wheal size and the number of positive SPT tests.

**Conclusion:**

This study highlights nutsedge, para grass, and Bermuda grass as major allergenic pollen sources for Thai AR patients. Including nutsedge, hurricane grass, and careless weed in clinical SPT panels is recommended for improved diagnostic accuracy. Additionally, the positive correlation between AR severity and pollen reaction strength emphasizes the importance of implementing patient education and avoidance strategies.

## 1 Introduction

### 1.1 The prevalence and impact of allergic rhinitis

Allergic rhinitis (AR) has become a significant public health issue worldwide, as its prevalence has been on the rise in recent decades (1). It is common for individuals with comorbidities such as asthma to also experience AR, which can result in reduced quality of life, academic or job-related performance, and significant financial strain (2). AR affects 10–20% of the population worldwide, particularly as much as 40% of the population in industrialized countries; and 45% of the population in Asia (3, 4). In Thailand, the prevalence of AR has been steadily increasing: a previous study showed that it rose from approximately 38 to 51% between 1995 and 2001 (5). Additionally, the prevalence of AR in Thai children (13–24%) is slightly higher compared to the Asia-Pacific region average (6–15%) and the global average (9–16%) (6).

### 1.2 Grass and weed pollen sensitization

Grass and weed pollen plays a key role as a major driver of allergic sensitization, with studies indicating that they are among the most common sources of AR, especially seasonal AR (SAR) (7, 8). These types of pollen are prevalent year-round in many parts of the world, especially in Southeast Asia, including Thailand (9, 10). Grass pollen sensitization is widespread in certain populations, with some studies reporting a prevalence as high as 70% (11–14). Seasonal grass pollen allergy typically occurs during spring and summer. In the general US population (10,348 studied subjects), approximately 25–27% and 18–22% of individuals were sensitized to rye and Bermuda grass pollen, respectively (15–17). In southern USA, such as south Florida, about 57% of AR patients were sensitized to Bahia grass (*Paspalum notatum* Flüggé) (18). In Europe, several grass pollen species are widely distributed in the atmospheric air, such as timothy grass (*Phleum pratense* L.), rye grass (*Secale cereale* L.), orchard grass (*Dactylis glomerata* L.), and meadow foxtail (*Alopecurus* spp.) (19). The prevalence of grass pollen allergy was almost 90% (49,910/55,661) among European patients diagnosed with allergic rhinoconjunctivitis (20). In most Western European countries, such as Belgium, France, Germany, Italy, Spain, and the UK, about 50% of AR patients are sensitized to grass pollen (21). In China, high percentages of up to 50 and 70% of AR patients are sensitized to Bermuda grass (*Cynodon dactylon* (L.) Pers.) and timothy grass (*Phleum pratense* L.) pollen allergens, respectively (22, 23). Among Thai AR patients, the main grass species that induce allergic sensitization include Bermuda grass (*Cynodon dactylon* (L.) Pers., ~17–52% of AR patients), para grass (*Urochloa mutica* (Forssk.) T.Q.Nguyen, ~50% of AR patients), Johnson grass (*Sorghum halepense* (L.) Pers., ~21% of AR patients), and Bahia grass (*Paspalum notatum* Flüggé, ~16% of AR patients) (5, 18, 24). Additionally, Manila grass (*Zoysia matrella* (L.) Merr.) and hurricane grass (*Bothriochloa pertusa* (L.) A.Camus) have recently been identified as species that induce sensitization in Thai AR patients (25).

Weed pollen is also a common causative allergenic source in several countries. In California, weeds are prevalent in almost 40% of patients with respiratory allergies (26). Ragweed (*Ambrosia* spp.) affects up to 15.3% of the general population in Northern and Central America (27, 28). Mugwort (*Artemisia vulgaris* L.) elicits allergic reactions in 10–14% of patients with pollinosis in Europe (29) and in 11.3% of patients with rhinitis and/or asthma in China (30). Additionally, *Amaranthus*, *Chenopodium*, and *Salsola* are significant sources for allergic reactions in temperate regions of Europe, in semi-desert regions of Iran, Kuwait, and Saudi Arabia, and in the western United States (29). Patients with allergic rhinitis (AR) in Thailand were found to be sensitized to careless weed (*Amaranthus hybridus* L.), sedge (*Carex* spp.), and cattail (*Typha latifolia* L.) pollen extracts (5, 31, 32). Moreover, careless weed pollen extracts induced positive skin prick test (SPT) reactivity in both Thai children with allergic respiratory diseases (33) and Thai adult patients with urticaria or allergic symptoms (34).

### 1.3 Co-sensitization and cross-reactivity

Co-sensitization among grass and/or weed pollen species is not uncommon, with studies reporting that up to 75–90% of patients with grass pollen allergy are also sensitized to weed pollen (8, 23). This evidence highlights the importance of identifying the specific aeropollen species that cause allergic sensitization in order to provide accurate diagnosis and treatment. However, it is essential to distinguish between co-sensitization and cross-reactivity—known as cross-sensitization. The failure to differentiate between these two phenomena may lead to misinterpretation of allergy test results and incorrect treatment decisions. The term “co-sensitization” is used to describe the state in which an individual has developed sensitization to multiple allergens that are not related to each other, while “cross-reactivity” refers to the phenomenon where IgE antibodies, which were initially generated against a particular allergen, bind to a structurally similar protein in another allergen (35, 36). In general, allergen cross-reactivity requires more than 70% identity in the amino acid sequence (35).

### 1.4 Geographical variations in allergen sensitization

Highly identical deduced amino acid sequences of group-1 grass pollen allergen have previously been reported in commonly found subtropical grasses, suggesting that these grasses could be cross-reactive (37). Meanwhile, cross-reactivity has been investigated among tropical/subtropical grass pollen allergens. For example, Uro m 1 has been shown to effectively inhibit Bermuda grass and Johnson grass pollen extracts (38). Additionally, Zoy m 1 showed significant cross-reactivity with Bermuda grass pollen extract (25).

The sensitization pattern of aeroallergens including grass and weed pollen varies across geographical regions because of differences in local climate, people lifestyle, and level of urbanization (27, 28). Update information regarding the offending aeroallergens in a local setting at a particular time is essential for effective managements of AR (32). However, in tropical/subtropical regions, the data about co-sensitization as well as cross-sensitization of grass and weed pollen is still scarcely available.

### 1.5 Research objectives and contributions

This study aimed to (1) evaluate the incidence and analyze the pattern of grass and weed sensitization in Thai AR patients and (2) determine the association between demographic characteristics and clinical data in these patients. This study represents a pioneering effort as it is the first to report patterns and correlations of sensitization to local grass and weed pollen species in Bangkok, Thailand. The results of this study provided essential information about grass and weed sensitization, as well as co-sensitization, which would be applied for guiding and improving the diagnosis of AR patients in Thailand and Southeast Asia.

## 2 Materials and methods

### 2.1 Pollen extracts

Grass and weed pollen extracts at 10,000 PNU/mL were prepared at the Department of Pharmacology, Faculty of Medicine Siriraj Hospital, Mahidol University in Bangkok, Thailand as described previously (39). Five grass pollen extracts from Bermuda grass (*Cynodon dactylon* (L.) Pers., Cd), para grass (*Urochloa mutica* (Forssk.) T.Q.Nguyen, Um), Johnson grass (*Sorghum halepense* (L.) Pers., Sh), Manila grass (*Zoysia matrella* (L.) Merr., Zm), and hurricane grass (*Bothriochloa pertusa* (L.) A. Camus, Bp), and two weed pollen extracts from nutsedge (*Cyperus mitis* Steud., Cm) and careless weed (*Amaranthus hybridus* L., Ah) were used in this study. Sh, Zm, and Bp pollen extracts were unavailable for testing on specific dates due to seasonal limitations and preparation requirements, leading to some participants not being tested with these pollen extracts. Additionally, recruiting participants for re-testing was difficult due to certain limitations.

### 2.2 Patients

This study was approved by the Siriraj Hospital Institutional Review Board (approval number: Si 171/2017). Thai adult patients (≥18 years old) diagnosed with allergic rhinitis (AR) were recruited for skin prick test (SPT). The subjects were recruited for this study from June 2017 to September 2020. AR was defined as a presence of two or more of the following symptoms: nasal itching, nasal congestion, rhinorrhea, and sneezing (40). Severity of AR based on patient symptoms was classified as mild intermittent, moderate to severe intermittent, mild persistent, and moderate to severe persistent, according to Allergic Rhinitis and its Impact on Asthma (ARIA) guideline (41). Before SPT, all patients signed an informed consent form. The recruited subjects were advised to discontinue antihistamine and/or intranasal corticosteroid use for at least 1 week prior to undergoing skin prick testing. Exclusion criteria included pregnancy, severe allergic reaction, history of anaphylaxis, chronic diseases, and skin lesion at test area.

### 2.3 Skin prick test

SPT was conducted by qualified technicians at the ENT Allergy clinic, Siriraj Hospital in Bangkok, Thailand. The SPT as the standard recommendation by GA^2^LEN guidelines was performed on the underside of the forearm by applying one drop of each pollen extract 3 cm apart (42). The skin was lightly pricked with a disposable 26-gauge needle in the center of each extract drop, using minimal force. Histamine and normal saline were applied as positive and negative controls, respectively. The threshold for SPT to be positive was wheal size of 3×3 mm or greater with concomitant flare. Among 121 recruited AR patients, patients with SPT positive to at least one pollen species extract were considered as a positive group, while patients with negative to all pollen extracts were regarded as a negative group.

### 2.4 Data processing and statistical analysis

Data extracted from medical records consisted of demographic data and SPT results. Demographic data: age, sex, environment, family history of allergic disease, age of onset, severity of AR, current medication, and comorbidities, are presented as mean ± standard deviation (SD) and range for continuous data, or frequency and percentage for categorical data. SPT results in response to grass and weed pollen extracts are presented as mean wheal diameter.

Data illustration and statistical analysis were performed using GraphPad Prism version 9.4.1 (GraphPad Software, CA, United States). The assumptions of normality and homogeneity of variance were tested using Shapiro–Wilk test and Levene’s test, respectively. For each pollen species, Mann–Whitney *U* test, a non-parametric test, was used to analyze whether there was a significant difference in the average diameter of SPT wheals between patients with AR symptoms manifesting before 20 years of age and those with a later onset (at or after 20 years). For all analyses, a *p*-value of less than 0.05 (*p* < 0.05) was considered statistically significant.

A Venn diagram was constructed to depict the co-sensitization of grass and weed pollen obtained from the SPT results of the patients using R version 4.2.2 (43), RStudio version 2022.12.0 + 353 (44), and the venn package version 1.11 (45).

Principal component analysis (PCA) was applied to identify patterns and associations of SPT data among the seven species of pollen extracts (Cd, Um, Sh, Zm, Bp, Cm, and Ah). The PCA scores were visualized using scatter plots for the four types of AR severity (mild intermittent, moderate to severe intermittent, mild persistent, and moderate to severe persistent), two types of comorbidities [without comorbidities (no) and with comorbidities (yes)], and two types of age of onset (< 20 and ≥ 20 years). Spearman’s rank correlation was applied in order to assess the correlation coefficient (r_s_) of the demographic and SPT data of the patients.

## 3 Results

### 3.1 Demographics

A total of 121 patients, who were diagnosed with allergic rhinitis (AR) and aged 18 years or above, were included in this study. The demographic and clinical characteristics of the patients are shown in Supplementary Table S1, and summarized in Table 1. Of the 121 patients, the majority were female (77/121, 63.6%). The average age of the patients was 34.2 ± 11.8 years, with a range of 18 to 69 years. The age group with the highest representation was 20 to 29 years old (52/121, 43%), followed by 30 to 39 years old (31/121, 25.6%). About 41.3% (50/121) of the patients reported having pets in their household, and only 9.1% (11/121) were smokers. Half of the patients (60/121, 49.6%) had no family history of allergies, while the most common family history reported was AR (48/121, 39.7%). The average age of onset of symptoms was 18.5 years, with a range of 0.5 to 59 years. The majority of patients had moderate to severe persistent symptoms (54/121, 44.6%), while 28.9% (35/121) had mild intermittent symptoms. Most patients were taking antihistamines (80/121, 66.1%), and 58.7% (71/121) were taking intranasal corticosteroids. A total of 40 patients (33.5%) reported having at least one comorbidity, with allergic rhinoconjunctivitis being the most common (25/121, 20.7%).

**Table 1:** Demographic and clinical characteristics of allergic rhinitis patients (*n* = 121).

Characteristics	*n*	%
**Gender**		
Male	44	36.4
Female	77	63.6
**Current age** (average ± SD: 34.2 ± 11.8, median: 31.0, range: 18–69)		
< 20 years	3	2.5
20–29 years	52	43.0
30–39 years	31	25.6
40–49 years	19	15.7
> 49 years	16	13.2
**Environmental conditions**		
Smoking	11	9.1
Pet in the household	50	41.3
**Family history with allergic diseases**		
No	60	49.6
Allergic conjunctivitis	1	0.8
Allergic rhinitis	36	29.8
Asthma	9	7.4
Food allergy	3	2.5
More than one allergic disease	12	9.9
**Age of onset** (average ± SD: 18.5 ± 15.4, median: 15.0, range: 0.5–59)		
< 1 year	4	3.3
1–9 years	40	33.1
10–19 years	20	16.5
20–29 years	29	24.0
30–39 years	14	11.6
> 39 years	14	11.6
**Severity symptoms**		
Mild intermittent	35	28.9
Moderate to severe intermittent	19	15.7
Mild persistent	13	10.7
Moderate to severe persistent	54	44.6
**Current medications**		
No	29	24.0
AH	21	17.4
IC	12	9.9
AH, IC	58	47.9
AH, IC, NI	1	0.8
**Comorbidities**		
No	81	66.9
Atopic dermatitis/atopic eczema	3	2.5
Allergic rhinitis	9	7.4
Allergic rhinoconjunctivitis	4	3.3
Asthma	1	0.8
Food allergy	2	1.7
Obstructive sleep apnea	5	4.1
Two comorbidities	11	9.1
More than two comorbidities	5	4.1

AH, antihistamine; IC, intranasal corticosteroid; LA, leukotriene antagonist; IT, immunotherapy.

### 3.2 Incidence of pollen sensitization

Five types of grass pollen (Bermuda grass, Cd; para grass, Um; Johnson grass, Sh; Manila grass, Zm; Hurricane grass, Bp) and two types of weed pollen (nutsedge, Cm; careless weed, Ah) were extracted for skin prick tests (SPT) (Supplementary Table S2). All 121 patients included in this study underwent SPT investigation to assess pollen sensitization, using the seven pollen extracts. Of these patients, 104 patients (85.95%) had a positive reaction to at least one pollen extract, while 17 patients (14.45%) were negative to all pollen extracts. No SPT data for some patients was available for Sh, Zm, and Bp extracts due to their unavailability on the test date.

**Figure 1:**
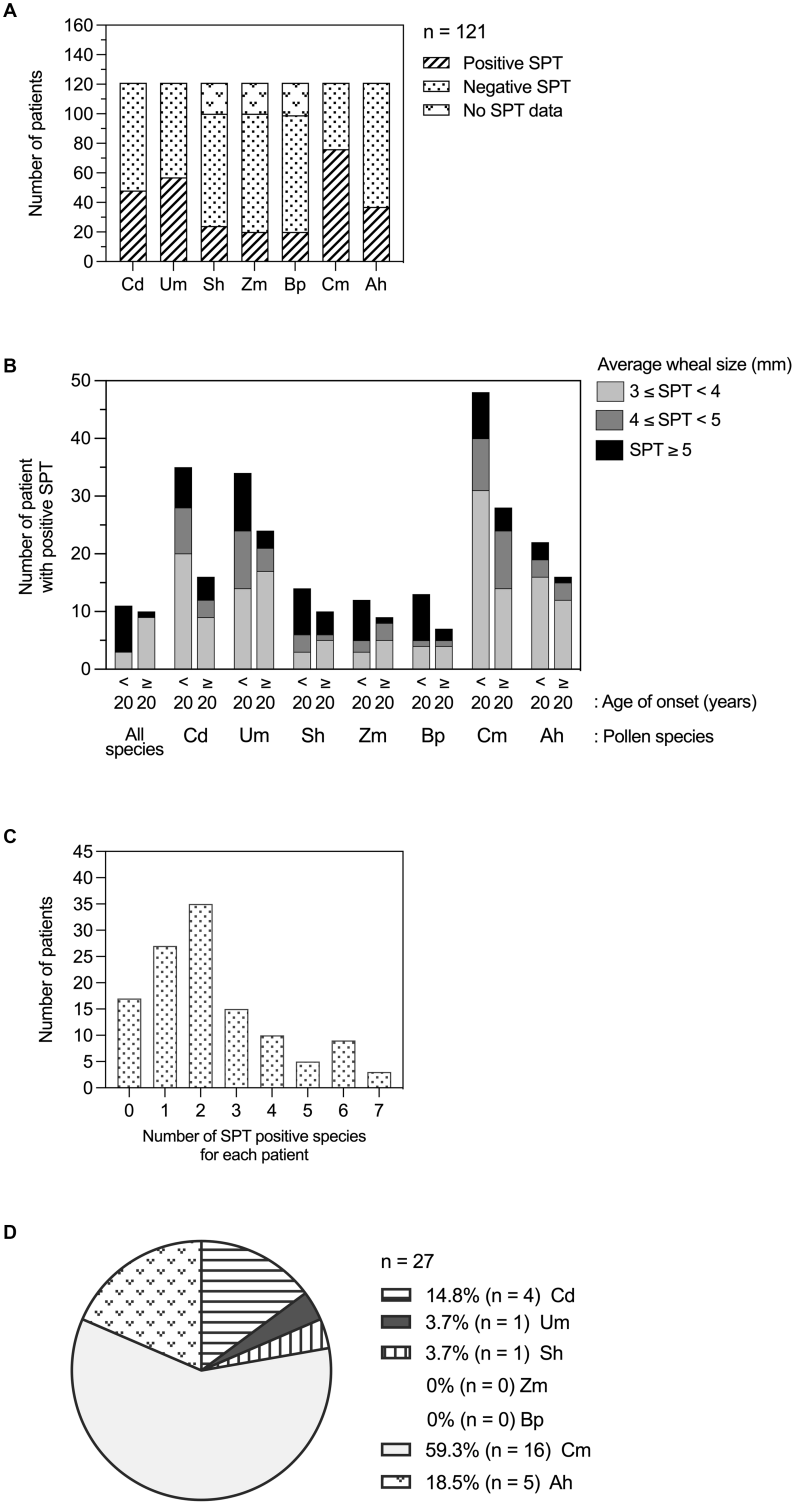
Allergic rhinitis patients with positive SPT results to grass and weed pollen extracts. **(A)** Patients with positive skin prick test to each species of grass and weed pollen extracts. **(B)** Number of patients with positive SPT for each grass and weed pollen species, categorized by average SPT wheal size and age of onset. **(C)** Number of patients in response to pollen extracts (number of species). **(D)** Proportion of patients with positive SPT to a single pollen species. A wheal size of 3 × 3 mm or greater on the SPT was determined as a positive result, while a wheal size of less than 3 mm was considered as a negative result. Patients without SPT data due to unavailable pollen extracts were identified as the “No SPT data” group. Cd, *Cynodon dactylon* (Bermuda grass); Um, *Urochloa mutica* (para grass); Sh, *Sorghum halepense* (Johnson grass); Zm, *Zoysia matrella* (Manila grass); Bp, *Bothriochloa pertusa* (hurricane grass); Cm, *Cyperus mitis* (nutsedge); Ah, *Amaranthus hybridus* (careless weed).

Prevalence of pollen sensitization varied by species with Um grass pollen extract having the highest number of positive SPT results (57/121), followed by Cd (48/121), Sh (24/121), Zm (20/121), and Bp (20/121). In weed pollen extracts, Cm had a higher number of positive SPT results (76/121) compared to Ah (37/121) (Figure 1A).

Analysis of the SPT wheal size revealed a probable tendency with age of onset. Patients who experienced AR symptoms before 20 years old (age of onset <20 years) exhibited larger wheal sizes compared to those whose symptoms began at 20 years or later (age of onset ≥20 years). This trend was evident in both the mean and median values across all pollen species tested (Supplementary Table S3). Furthermore, the prevalence of patients with wheal sizes exceeding 5 mm in response to all pollen species was likely higher in the younger age of onset group. This pattern held true for individual pollen types, specifically those tested with Cd and Um extracts (Figure 1B).

### 3.3 Co-sensitization patterns

Among 104 AR patients with positive SPT, the most common poly-sensitization (35/104) showed sensitivity to two different pollen extracts (Figure 1C). Of the 27 mono-sensitized patients, six (6/27, 22.2%) and 21 (21/27, 77.8%) patients were sensitized to grass and weed pollen extracts, respectively. Among these patients, several patients were sensitized to Cm (16/27, 59.3%), Ah (5/27, 18.5%), and Cd (4/27, 14.8%) pollen extracts, while only one patient was singly sensitized to Um (1/27, 3.7%), and Sh (1/27, 3.7%) pollen extracts (Figure 1D).

**Figure 2:**
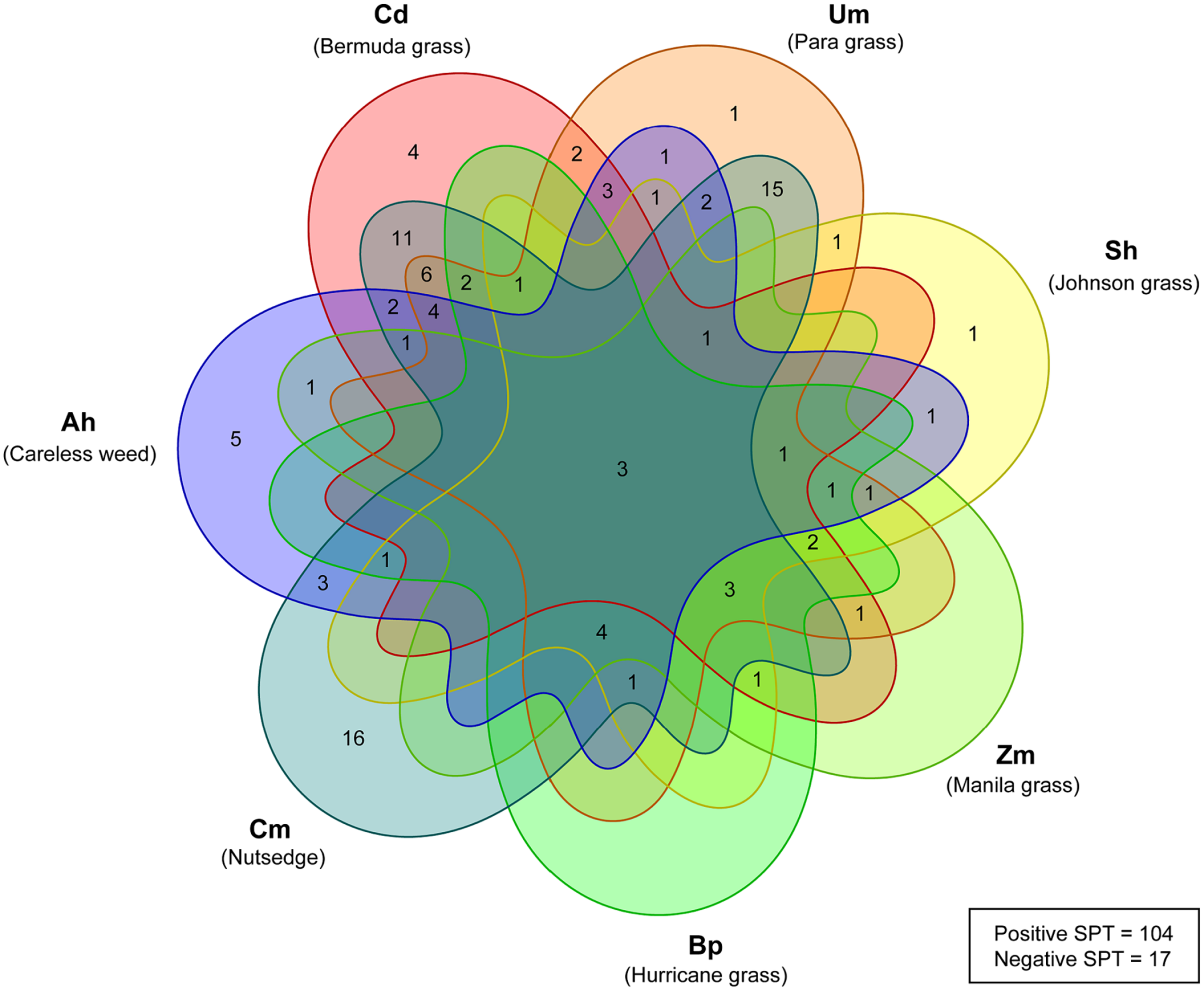
Venn diagram of grass and weed sensitization among Thai allergic rhinitis patients based on SPT data. Empty region represents no reported patient in that segment. A wheal size of 3 × 3 mm or greater on the SPT was determined as a positive result, while a wheal size of less than 3 mm was considered as a negative result. Patients without SPT data due to unavailable pollen extracts were identified as the “No SPT data” group. Out of the 104 patients responding positively to at least one pollen extract, 35 (33.7%), 15 (14.4%), 10 (9.6%), 5 (4.8%), 9 (8.7%), and 3 (2.9%) exhibited positive SPT responses to 2, 3, 4, 5, 6, and 7 pollen extracts, respectively. Notably, Cm showed a considerably higher prevalence of sensitization (16/104) compared to other single pollen extracts. Poly-sensitization patterns were also observed, with Um-Cm being the most frequent (15/104 patients), followed by Cd-Cm (12/104 patients). Moreover, only three patients (3/104) displayed positive SPTs to all seven tested pollen extracts.

The Venn diagram presented in Figure 2 illustrates the co-sensitization patterns of grass and weed pollen among patients diagnosed with allergic rhinitis (AR). Out of the 104 patients who exhibited a positive response to at least one pollen extract, 35 (33.7%), 15 (14.4%), 10 (9.6%), 5 (4.8%), 9 (8.7%), and 3 (2.9%) patients showed positive SPT reactions to 2, 3, 4, 5, 6, and 7 pollen extracts, respectively (Figures 1C, 2). Of these patients, the prevalence of sensitization was considerably high for Cm at 16/104 compared to other single pollen extracts. For poly-sensitization, Um-Cm was the most frequently observed co-sensitization pattern, with 15/104 patients, followed by Cd-Cm, with 11/104 patients. Only three patients (3/104) showed positive SPTs to all seven pollen extracts tested.

The number and percentage of patients with co-sensitization to two pollen species are presented in Table 2. Overall, a substantial percentage of co-sensitizations was observed in Cm or Bp with other species. Specifically, more than 70% of the patients showed co-sensitization between Cm and Cd (70.8%), Um (73.7%), or Bp (75.0%). Additionally, 75% of the patients co-sensitized to Bp and Sh or Zm, while about 70% of patients co-sensitized to Sh and Zm.

**Table 2:** Co-sensitization of grass and weed pollen species.

Species 1	Species 2	*n*	%
Cd (Bermuda grass) (*n* = 48)	Um	29	60.4
	Sh	12	25.0
	Zm	13	27.1
	Bp	13	27.1
	**Cm**	**34**	**70.8**
	Ah	15	31.3
Um (Para grass) (*n* = 57)	Sh	20	35.1
	Zm	17	29.8
	Bp	18	31.6
	**Cm**	**42**	**73.7**
	Ah	23	40.4
Sh (Johnson grass) (*n* = 24)	**Zm**	**17**	**70.8**
	**Bp**	**18**	**75.0**
	Cm	14	58.3
	Ah	15	62.5
Zm (Manila grass) (*n* = 20)	**Bp**	**15**	**75.0**
	Cm	12	60.0
	Ah	13	65.0
Bp (Hurricane grass) (*n* = 20)	**Cm**	**15**	**75.0**
	Ah	11	55.0
Cm (Nutsedge) (*n* = 76)	Ah	22	28.9

Bold letters indicate a co-sensitization rate of more than 70%. “*n*” represents the number of patients with positive SPT reaction. The percentage of co-sensitization of weed and grass pollen species was calculated by dividing the number of patients with a positive SPT reaction to pollen Species 2 by the number of patients sensitized to pollen Species 1, expressed as a percentage.

### 3.4 SPT wheal size patterns

**Figure 3:**
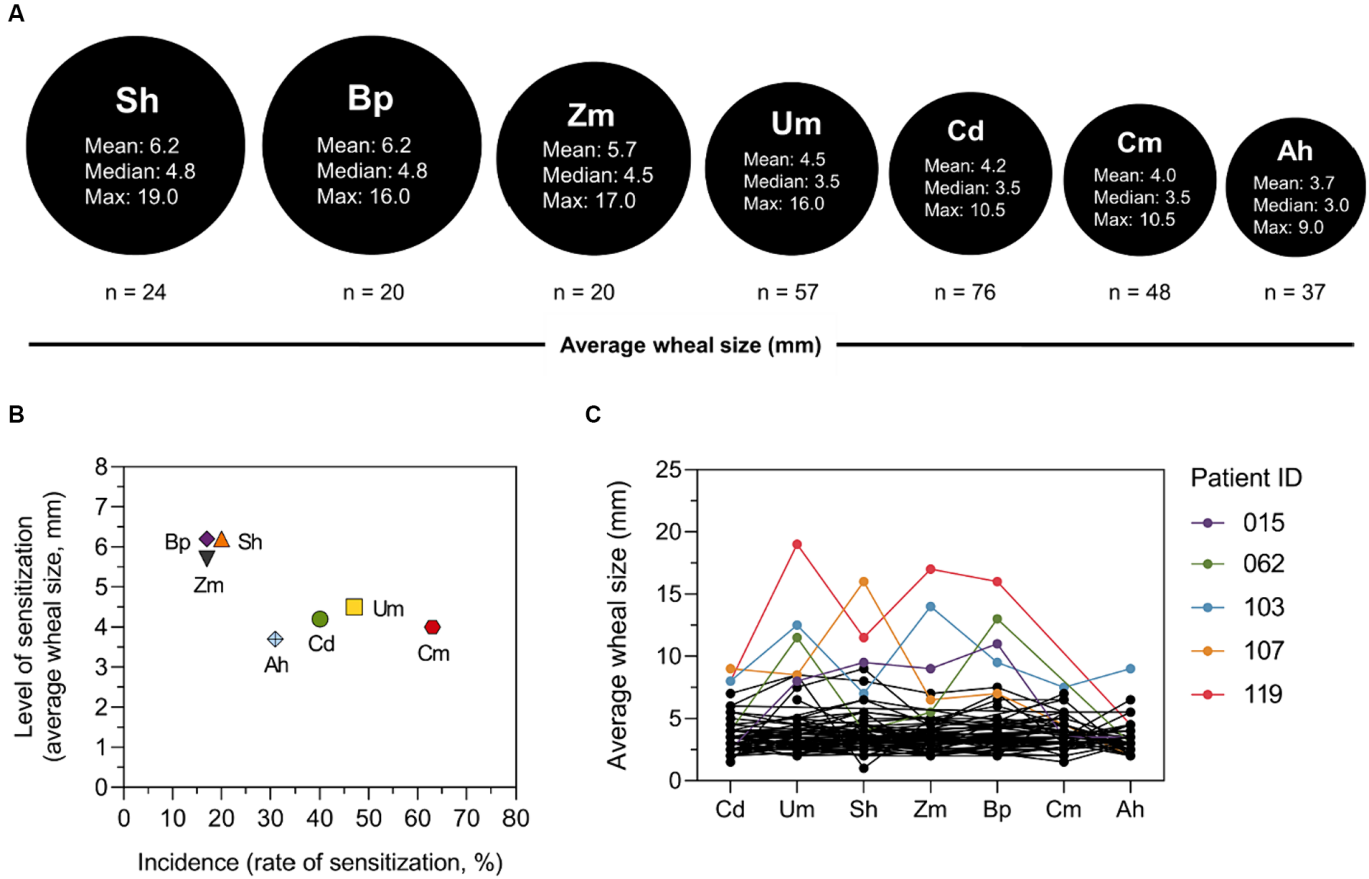
Average SPT wheal size of patients in response to grass and weed pollen extracts. **(A)** The mean, median, and maximum average wheal size of patients to each pollen species. **(B)** Scatter plot illustrating level and rate of sensitization for each species. **(C)** Wheal size of individual patient to each pollen species. Patterns of average wheal size of individual point across all species. Patients with the largest wheal size for at least one species were plotted in colors.

The average SPT wheal size of patients responding to grass and weed pollen extracts is illustrated in Figure 3. The mean and median values are shown, as well as the maximum value observed (Figure 3A). The mean of wheal size induced by Bp and Sh extracts were the largest (6.2 mm), compared to other species. The median wheal size for Sh, Bp, and Zm ranged from 4.5 mm to 4.8 mm, whereas Um, Cd, Cm, and Ah exhibited sizes ranging from 3.0 mm to 3.5 mm. The maximum wheal size ranged from 19 mm for Sh to 9 mm for Ah. The relationship between level of sensitization and incidence is illustrated in Figure 3B. Level of sensitization refers to the average SPT wheal size elicited by each pollen species, and incidence is determined by rate or frequency of patient sensitization to each pollen species. Of the total 121 patients, the highest incidence was sensitization to Cm, while level of Cm sensitization was relatively low. The highest level of sensitization was induced by Sh and Bp; however, their incidence was low. The SPT wheal size of individual patient to each pollen species is shown in Figure 3C. Overall, individuals with extremely high SPT wheal size for one species were likely to have high SPT wheal for other, although not all, species. For instance, patient no. 119 had the largest wheal size for 3/7 species.

### 3.5 SPT results and AR symptom severity

**Figure 4:**
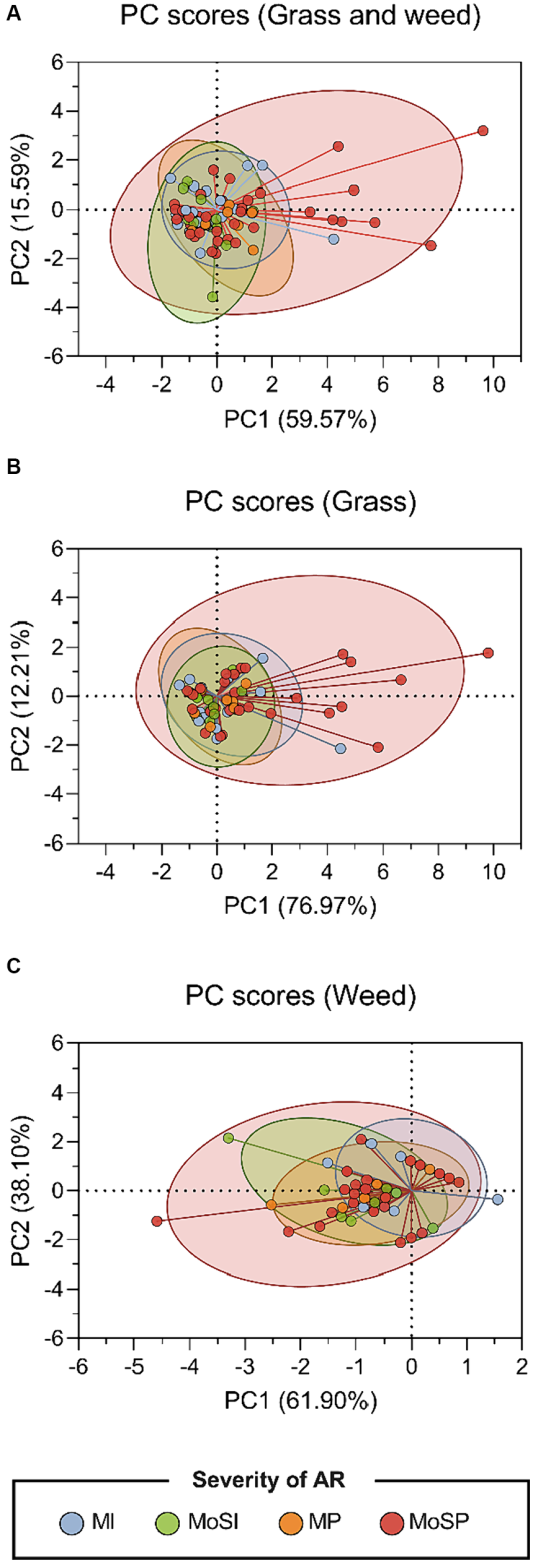
Principal component analysis of SPT results for all pollen species. Scatter plots of the two-dimensional principal component analysis (PCA) illustrates the elements of the linear combinations (PC1 score in X-axis and PC2 score in Y-axis) for the SPT data in response to **(A)** all pollen species, **(B)** grass species, and **(C)** weed species. The coordinates were represented in different colors, indicating the group of AR severity: mild intermittent (MI), moderate to severe intermittent (MoSI), mild persistent (MP), and moderate to severe persistent (MoSP).

Principal component analysis (PCA) was conducted to assess whether skin prick test (SPT) wheal size, and its combined effect with the number of positive SPTs, could differentiate groups defined by allergic rhinitis (AR) severity, age of onset, and comorbidity. SPT data were transformed into two principal components (PC1 and PC2) capturing the majority of data variability. PCA scatter plots (Figure 4) visualized the distribution of SPT data points and their association with distinct AR severity categories: mild intermittent (MI), moderate-to-severe intermittent (MoSI), mild persistent (MP), and moderate-to-severe persistent (MoSP). However, the plots revealed no clear separation between the four AR severity groups for either grass and weed pollen species (Figure 4A; Supplementary Figure S1A) or when analyzed separately for each (Figures 4B,C). Similarly, no distinct separation was observed between groups defined by age of onset (< 20 vs. ≥ 20 years) and comorbidity (presence or absence) (Supplementary Figures S1B,C). Furthermore, combining SPT wheal size with the number of positive SPTs in the PCA did not reveal separation by AR severity, age of onset, or comorbidity, suggesting that SPT wheal size remains consistent across these groups regardless of the number of positive tests (Supplementary Figures S1D–F).

### 3.6 Relationship between SPT wheal size and sensitization

**Figure 5:**
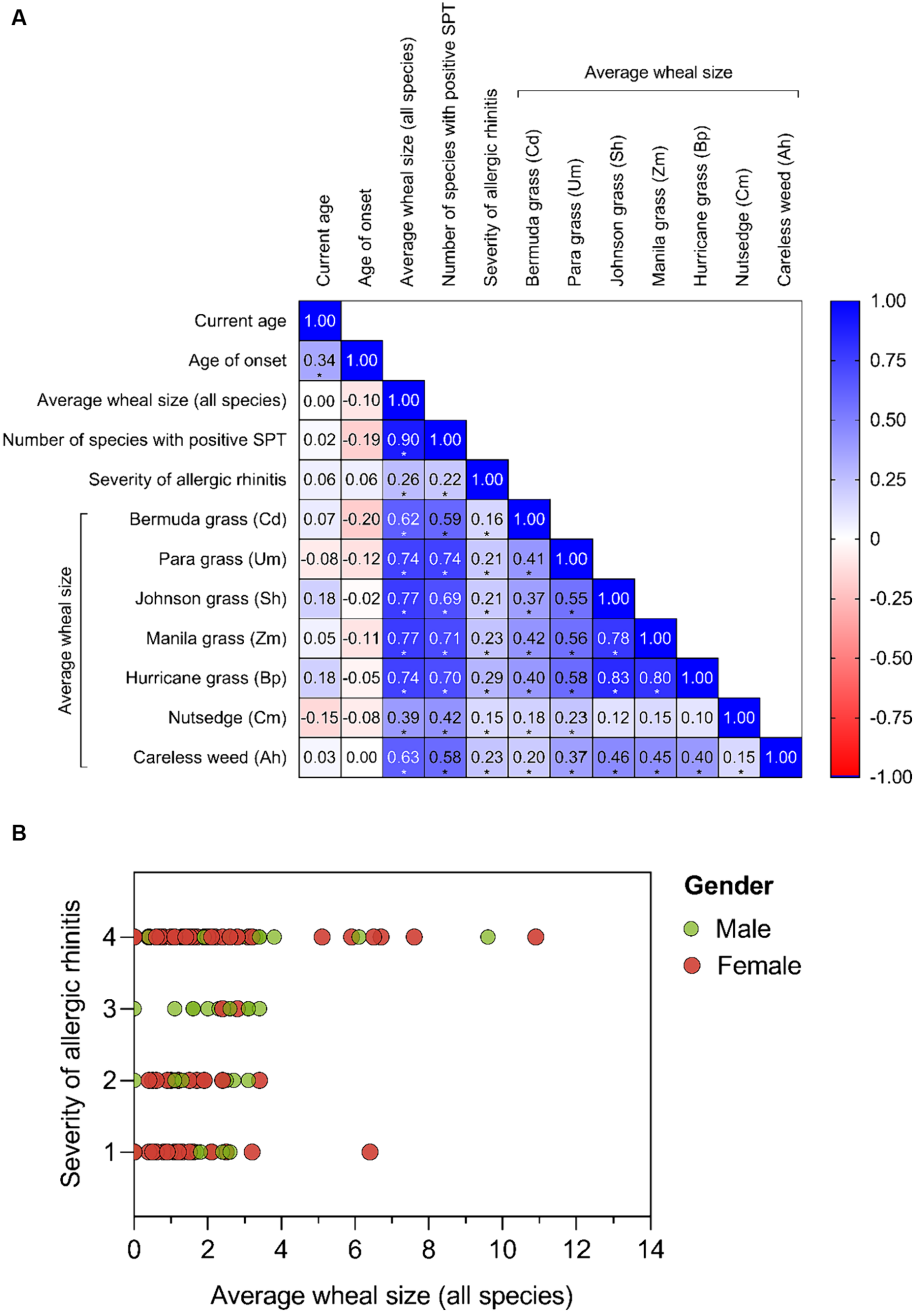
Correlation analysis of demographic and clinical characteristics of AR patients. **(A)** The heat map presents the pairwise correlation between analyzed factors, including current age, age of onset, number of species with positive SPT, severity of AR, and average SPT wheal size in response to grass and weed pollen extracts. The map includes Spearman’s correlation coefficients and indicates statistically significant correlations with an asterisk (^*^) at a significant level of 0.05. **(B)** The scatter plot depicts the relationship between the severity of AR (rated as 1: mild intermittent, 2: moderate to severe intermittent, 3: mild persistent, and 4: moderate to severe persistent) and the average wheal size in response to all pollen extracts.

There was a highly significant and strong positive correlation (r_s_ = 0.90; *p* < 0.0001) between the average SPT wheal size of all pollen species and the number of pollen species with a positive SPT result. The average wheal size of all pollen species also had a weak correlation (r_s_ = 0.26, *p* = 0.004) with the severity of AR symptoms (Figures 5A,B). Additionally, the severity of AR symptoms was correlated with the number of species that elicited positive SPT result in a given individual (r_s_ = 0.22, *p* = 0.017) (Figure 5A). The average wheal size of all pollen species and the number of species with a positive SPT result had a strong positive correlation with the average wheal size of individual pollen species, except for Cm pollen, which showed only a moderate correlation (Figure 5A). Furthermore, most patients with the average wheal size of more than 4 mm experienced moderate to severe persistence. However, some patients with moderate to severe persistence exhibited a low average SPT wheal size. The majority of patients with mild persistence were male (Figure 5B).

Regarding individual pollen species, the average wheal size of SPT in response to Cd pollen had a moderate positive correlation with all grass pollen species and a weak correlation with all weed pollen species (Figure 5A). Um pollen showed a moderate correlation with most pollen species, except for Cm pollen, which showed only a weak correlation. Sh and Zm had a strong correlation with Bp (Sh-Bp: r_s_ = 0.83, *p* < 0.0001; Zm-Bp: r_s_ = 0.80, *p* < 0.0001) and a moderate correlation with Ah (Sh-Ah: r_s_ = 0.46, *p* < 0.0001; Zm-Ah: r_s_ = 0.45, *p* < 0.0001). The correlation between the average wheal size of Ah pollen and the average wheal size of Bp and Cm pollen was moderate (r_s_ = 0.40, *p* < 0.0001) and very weak (r_s_ = 0.15, *p* < 0.097), respectively.

## 4 Discussion

In recent decades, the incidence of allergic rhinitis (AR) has increased progressively due to various factors such as climate change, urbanization, economic growth, and changes in dietary habits towards a more Westernized style (21, 46–48). Identifying the allergens responsible for triggering this allergic disease is crucial for diagnosis and prevention. Moreover, investigating allergens is necessary for allergic immunotherapy, which is fundamental in treating AR. Wind-pollinated pollen produced by different plant species is one of the most significant outdoor sources of allergens that can harm atopic individuals, especially those with AR and asthma. Among the airborne pollen species found in the atmosphere, grass and weed pollen have been reported as major culprits of allergic reactions in AR patients. However, common pollen allergens that cause allergic reactions can differ greatly by country or region (49).

Data on sensitization and co-sensitization of grass and weed pollen are scarce in Southeast Asia, possibly leading to patients being misdiagnosed or underdiagnosed in the region. This study demonstrates the patterns of sensitization and co-sensitization of common grasses and weeds among patients with AR in Bangkok, Thailand. A total of 121 AR patients 18 years or older, most of whom had a positive SPT to at least one type of pollen, were included in the study. There were a few limitations of this study, which limited the number of cases to only 121, from only one region in the country. First, the inclusion criteria required patients to be sensitized to at least one grass or weed pollen species based on SPT that must be administered by allergy specialists in specified allergy clinics. These clinics are concentrated within the Bangkok metropolitan areas. Thus, patients from other regions often get referrals to major hospitals in Bangkok to be tested. Since AR is not life-threatening, patients often choose not to get tested. Secondly, grass and weed pollen sensitization is presumably under-diagnosed in this country because the pollen season is not obvious, leading to fewer patients seeking medical diagnostics. Third, of the patients seeking medical diagnostics, very few were tested using extracts from local species. The two pollen species with commercially available extracts are *Cynodon dactylon* (Bermuda grass) and *Sorghum halepense* (Johnson grass). The extracts of different grass pollen species used in this study were produced in-house in limited quantities and could not be distributed to other allergy clinics for testing. To address these limitations and gain a more comprehensive understanding of AR in Thailand, future research should pursue several key directions. First, expanding the geographical scope of investigations is crucial. Conducting studies in diverse regions beyond Bangkok will capture a more representative national picture of AR prevalence and facilitate the identification of regional variations in pollen sensitization patterns. Second, a comprehensive evaluation of the impact of less-recognized pollen seasons is imperative. Thorough collection and analysis of year-round pollen count data can establish a more precise understanding of pollen seasonality across diverse regions in Thailand. This includes identifying peak periods for various grass and weed species. Correlating this data with reported AR symptom patterns will facilitate the assessment of potential links between less-recognized pollen seasons and the under-diagnosis of AR. Finally, incorporating local pollen species into routine diagnostic practices is essential. Future research should advocate for the development and commercialization of standardized allergen extracts from prevalent local species, such as *Cyperus mitis* (nutsedge). Additionally, encouraging the inclusion of these local extracts in routine allergy testing protocols has the potential to significantly improve diagnostic accuracy.

Females accounted for over half of the total AR patients, consistent with previous meta-analysis studies that reported a shift in AR prevalence from a male predominance to a female predominance after puberty (50, 51). Similar trends were also observed in the Global Asthma Network (GAN) Phase I study—a cross-sectional, multicenter, international, epidemiological study, which reported a significantly higher prevalence of AR among female adolescents compared to males in Mexico (52). Over one-third of the AR patients in our study were between the ages of 20 and 29 years, many of whom reported developing symptoms of AR between the ages of 1 and 9. This finding is in accordance with previous epidemiological reports indicating that AR symptoms often develop before the age of 20 and are commonly found in individuals aged 20–40 years (53–55). Additionally, this study revealed a noteworthy association between the age of AR onset and SPT wheal size. Patients with earlier onset (< 20 years old) consistently exhibited larger wheal sizes across all pollen species tested, suggesting a potentially stronger allergic response. While the exact mechanisms require further exploration, this finding has potential clinical implications.

Approximately half of the study participants suffered from moderate to severe persistent symptoms, substantiated by the fact that most were taking antihistamines and/or intranasal corticosteroids as a general medication to alleviate their AR symptoms. AR is generally associated with comorbidities, particularly asthma. A meta-analysis revealed a prevalence of AR with asthma in China to be 10.17% (56), while a study in Bangkok, Thailand, reported that 27.1% of AR children also had asthma (57). However, more than half of the patients in our study did not exhibit any comorbidities. Among those, allergic rhinoconjunctivitis was the most prevalent, with asthma showing a prevalence of 2.5%. This study did not have specific tests for diagnosing asthma, potentially missing some patients who have the condition but remain undiagnosed. Despite most of the recruited patients lacking a family history of allergic diseases, almost 40% reported a family history of AR, suggesting a potential genetic predisposition to the disease. Additionally, although many patients had no family history of allergies, their AR development may have been influenced by environmental factors such as pet ownership. The demographic data revealed that 41.3% of the patients had a pet in their household. A previous study conducted in China found that pet ownership was associated with an increased risk of respiratory morbidities, such as chronic bronchitis and asthma (58). However, recent updates suggest that pet ownership could potentially alleviate the detrimental effects of prolonged air pollution on childhood asthma (59). Early-life exposure to dogs and cats might be protective against developing allergies and asthma in children (60) Nonetheless, the relationship between pet ownership and allergies/allergic rhinitis is complex and not fully understood. Another contributing environmental factor could be environmental pollution, especially air pollution and PM 2.5, which has been dramatically increased in the recent years in the region (61, 62).

This study explicates the current situation concerning sensitization to common grass and weed pollen among AR patients in Bangkok, Thailand. Among seven species of grass and weed species investigated, nutsedge, para grass, and Bermuda grass were the top three pollen species with the highest sensitization rates. These results are consistent with previous studies, which also implicated these three pollen species as one of the major triggers of allergic symptoms in AR patients (5, 32). Based on an airborne pollen study conducted in Bangkok, a bustling metropolis in Thailand, it was discovered that grass and weed pollen were pervasive in the atmospheric air all year round, with grass pollen being the most dominant in terms of concentration, closely followed by weed pollen (9, 10).

We observed a highly significant positive correlation between the average SPT wheal size and the number of pollen species eliciting positive SPT results. In addition, a positive correlation was observed between the average wheal size for each pollen extract and the severity of AR symptoms. This finding suggests that patients who were sensitized to a greater number of pollen species were more likely to have a larger SPT wheal size, and experience a stronger allergic reaction. Our results aligned with a prior investigation, which demonstrated that patients, particularly adults, with multiple allergic sensitizations were significantly more likely to have severe rhinitis and asthma (63). Several prior studies also found a positive association between patient-reported clinical symptoms and positive reactions in SPT and levels of serum specific immunoglobulin E (sIgE) upon exposure to indoor/outdoor inhalant allergens like grasses, mites, and animal dander (63–67). Another study also underscored the distinct features of AR in poly-sensitized patients and mono-sensitized patients, prompting the need for classification of these patients into separate categories (68). Although our attempts to primarily distinguish patients using principal component analysis (PCA) of SPT results did not show a clear segregation of patients based on AR symptom severity, possibly due to insufficient information and further studies are needed to better understand features of grass-allergic AR patients in this region. Further studies should broaden their scope to investigate IgE reactivity, elucidating its presence and patterns for each pollen species. Additionally, efforts should be directed towards identifying both major and minor allergenic proteins, encompassing an exploration of cross-reactivity.

Pollen from grass species is generally understood to be extensively cross-reactive, as also supported by this study that the majority of patients were sensitized to more than one grass species. In fact, no patient was found to be mono-sensitized to hurricane grass nor Manila grass. On the other hand, the cross-reactivity was neither reciprocally equal nor complete. While six patients were monosensitized to a specific grass pollen, nine patients were sensitized to all five species of grasses. Non-reciprocal cross-reactivity of grass pollen has been reported previously (69). Bermuda grass pollen was the most unique of all five grasses. Intriguingly, Manila grass sensitization profile was close to that of hurricane grass, even though Manila grass and Bermuda grass belong to the same subfamily (Chloridoideae). Meanwhile, hurricane grass belongs to the same subfamily with the other two species (para grass and Johnson grass; subfamily Panicoideae).

Although no patient was found to be mono-sensitized to hurricane grass or Manila grass, these species could not be neglected because hurricane grass and Manila grass not only exhibited high frequencies of co-sensitization, but also high levels of sensitization as indicated by large average wheel size. In reality, AR symptoms could be triggered by a combination of pollen from a number of species, including those that have not been investigated. Further studies are needed to comprehend the composition, dynamics, and potency of airborne pollen in this region for better AR management, perhaps encompassing more airborne pollen surveys, molecular taxonomy, as well as field studies.

Our investigation also uncovered that the highest incidence of mono-sensitization was found with nutsedge and careless weed. This is likely because these species are less closely related to other grass species. Closely related species in the phylogenetic relationship tend to possess similar protein sequences, suggesting the possibility of IgE cross-reactivity (70). According to the Angiosperm Phylogeny Group (APG) classification of flowering plants, nutsedge is a member of the family Cyperaceae, in the same order (Poales) as grasses (family: Poaceae) (71). Whereas, careless weed (family: Amaranthaceae) is classified in a separate order with a more distant relationship (71). Currently nutsedge and careless weed are not included in the standard clinical practice for SPT in this region. In light of these results, it is strongly recommended that standard routine clinical allergy tests for pollen sensitization, including SPT, should encompass nutsedge, hurricane grass, and careless weed to ensure a comprehensive and accurate diagnosis.

It is crucial to recognize that the clinical severity of AR is multifaceted and can be influenced by various environmental and individual factors. As such, it is imperative to conduct further research to comprehend the complex interplay between these factors and their roles in the development and management of AR. Based on our investigation, clinical data, specifically the size of SPT wheal after being tested with pollen extracts, plays a crucial role in determining the development of AR symptoms. It can also serve as an indicator for AR patients to be cautious in exposure to allergenic pollen and take necessary preventive measures. Additionally, these findings underscore the significance of identifying and testing multiple pollen species in AR patients to develop tailored treatment plans, allowing for more personalized treatment strategies that target the specific allergens. This approach could potentially reduce the overall burden of AR symptoms and ultimately lead to improved patient outcomes and quality of life.

## 5 Conclusion

Our findings indicate that nutsedge, para grass, and Bermuda grass are the primary pollen sources responsible for causing allergic sensitization in Thai individuals with allergic rhinitis (AR). This study suggested that standard clinical allergy tests should encompass nutsedge, hurricane grass, and careless weed to ensure precise diagnosis, with substantial implications for understanding sensitization patterns and its extent. Additionally, the intensity of allergic responses to pollen is directly linked to the severity of AR symptoms, underscoring the importance of being cautious and adopting preventive measures. This study furnishes valuable insights for clinicians, scientists, and policymakers, aiding in the enhancement of diagnosis and management of pollen-induced allergies in Southeast Asia and other subtropical regions of the world.

## Data availability statement

The original contributions presented in the study are included in the article/Supplementary material, further inquiries can be directed to the corresponding author.

## Ethics statement

The studies involving humans were approved by Siriraj Hospital Institutional Review Board. The studies were conducted in accordance with the local legislation and institutional requirements. The participants provided their written informed consent to participate in this study.

## Author contributions

SA-i: Data curation, Formal analysis, Investigation, Methodology, Visualization, Writing – original draft, Writing – review & editing. YJ: Data curation, Formal analysis, Investigation, Methodology, Software, Visualization, Writing – original draft, Writing – review & editing. BP: Investigation, Methodology, Resources, Writing – review & editing. KT: Investigation, Methodology, Resources, Writing – review & editing. PT: Formal analysis, Visualization, Writing – original draft, Writing – review & editing. WS: Conceptualization, Formal analysis, Funding acquisition, Methodology, Supervision, Visualization, Writing – original draft, Writing – review & editing.
