# Supplementary material for: Incidence of grass and weed sensitization in Bangkok, Thailand: a clinical study
Source: Front Public Health. 2024 Mar 28;12:1301095. doi: 10.3389/fpubh.2024.1301095 (PMC11007029; doi:10.3389/fpubh.2024.1301095)
Supplement: Supplementary file 1 [file Data_Sheet_1.PDF]

Supplementary Material

Incidence of grass and weed sensitization in Bangkok, Thailand: a clinical study

Sirirat Aud-in[1,2†], Yotin Juprasong[2,3,4,7†], Bannapuch Pinkaew[6], Kanokporn Talek[6], Pongsakorn Tantilipikorn[5,6], and Wisuwat Songnuan[1,2,4,5*]

[1]Department of Plant Science, Faculty of Science, Mahidol University, Bangkok, Thailand

[2]Systems Biology of Diseases Research Unit, Faculty of Science, Mahidol University, Bangkok, Thailand

[3]Graduate Program in Toxicology, Faculty of Science, Mahidol University, Bangkok, Thailand

[4]Center of Excellence on Environmental Health and Toxicology (EHT), Office of the Permanent Secretary (OPS), Ministry of Higher Education, Science, Research and Innovation (MHESI), Bangkok, Thailand

[5]Center of Research Excellence in Allergy and Immunology, Faculty of Medicine Siriraj Hospital, Mahidol University, Bangkok, Thailand

[6]Division of Rhinology and Allergy, Department of Otorhinolaryngology, Faculty of Medicine Siriraj Hospital, Mahidol University, Bangkok, Thailand

[7]Department of Biochemistry, Faculty of Medicine, Srinakharinwirot University, Bangkok, Thailand

[†]**These authors contributed equally to this work and share first authorship**

[*]**Correspondence:** Wisuwat Songnuan; wisuwat.son@mahidol.edu

Supplementary Table 1 Demographic and clinical characteristics of allergic rhinitis (AR) patients (n=121).

		Patient ID	001	002	003	004	005	006	007	008	009	010	011	012	013	014	015	016	017	018	019
Demographic data		Age (years)	54	23	31	21	21	30	20	23	31	26	23	38	47	24	23	31	20	31	24
		Sex	Male	Male	Female	Male	Female	Male	Female	Male	Female	Female	Female	Female	Female	Male	Female	Female	Male	Female	Female
	Environmental conditions	Smoking	Yes	No	No	No	No	Yes	No	No	No	No	No	No	Yes	No	No	No	No	No	No
		Pet	No	No	No	No	No	No	No	No	Yes	Yes	Yes	No	Yes	No	No	No	No	No	Yes
		Family history with AD	No	Asthma	AR	No	No	No	AR<br>FA	No	No	No	AR	AR<br>FA	No	Asthma<br>AR	AR	AR	AR	No	AR
Allergic rhinitis data (Clinical data)		Age of onset (years)	10	3	10	3	5	5	9	15	1	3	4	23	20	10	10	8	13	4	24
		Severity of AR	MoSI	MI	MoSP	MoSP	MI	MI	MoSP	MI	MI	MI	MoSI	MoSP	MI	MoSP	MoSP	MoSP	MoSP	MoSI	MI
		Current medication	AH<br>IC	IC	No	IC	No	AH<br>IC	AH<br>IC	AH<br>IC	No	No	IC	AH<br>IC	IC	AH<br>IC	AH<br>IC	AH<br>IC	AH<br>IC	IC	No
		Comorbidities	ANM<br>CRR	No	No	No	No	No	No	No	No	No	No	AD/AE<br>FA	Asthma	No	No	ARC	No	No	No
	Skin prick test wheal diameter (d), d_{max} x d_{min} (mm)	Bermuda grass (Cd)	0<br>0	3<br>3	2<br>3	4<br>4	0<br>0	2<br>3	2<br>3	2<br>2	0<br>0	3<br>3	3<br>3	3<br>5	5<br>6	2<br>3	2<br>3	4<br>5	0<br>0	0<br>0	0<br>0
		Para grass (Um)	3<br>3	4<br>5	3<br>4	0<br>0	3<br>3	2<br>3	3<br>4	0<br>0	3<br>4	3<br>3	0<br>0	5<br>8	3<br>4	2<br>3	9<br>10	5<br>6	4<br>5	4<br>5	3<br>3
		Johnson grass (Sh)	3<br>4	2<br>2	0<br>0	0<br>0	0<br>0	0<br>0	0<br>0	3<br>3	0<br>0	0<br>0	0<br>0	0<br>0	0<br>0	3<br>4	6<br>10	0<br>0	3<br>5	0<br>0	0<br>0
		Manila grass (Zm)	3<br>3	2<br>2	0<br>0	0<br>0	0<br>0	0<br>0	2<br>3	2<br>3	0<br>0	0<br>0	0<br>0	0<br>0	0<br>0	2<br>2	8<br>10	0<br>0	0<br>0	0<br>0	0<br>0
		Hurricane grass (Bp)	3<br>3	0<br>0	0<br>0	0<br>0	0<br>0	0<br>0	0<br>0	3<br>4	0<br>0	0<br>0	0<br>0	0<br>0	0<br>0	2<br>3	10<br>12	0<br>0	2<br>3	0<br>0	0<br>0
		Sedge (Cm)	3<br>4	3<br>4	3<br>3	5<br>5	4<br>6	3<br>4	5<br>9	3<br>3	3<br>3	0<br>0	3<br>3	4<br>4	0<br>0	2<br>3	3<br>4	0<br>0	0<br>0	3<br>3	6<br>7
		Careless weed (Ah)	3<br>3	0<br>0	0<br>0	0<br>0	0<br>0	0<br>0	0<br>0	3<br>4	0<br>0	0<br>0	2<br>3	2<br>3	0<br>0	3<br>4	3<br>4	3<br>3	0<br>0	4<br>5	2<br>2

Abbreviations: AC, Allergic conjunctivitis; AD/AE, Atopic dermatitis/atopic eczema; ANM, Anosmia; AR, Allergic rhinitis; ARC, Allergic rhinoconjunctivitis; CRR, Chronic/recurrent rhinosinusitis; FA, Food allergy; OSA, Obstructive sleep apnea; MI, Mild intermittent; MoSI, Moderate to severe intermittent; MP, Mild persistent; MoSP, Moderate to severe persistent; AH, Antihistamine; IC, Intranasal corticosteroid; LA, Leukotriene antagonist; IT, Immunotherapy; n.d., No data.

Supplementary Table 1 Demographic and clinical characteristics of allergic rhinitis (AR) patients (n=121). *(cont.)*

		Patient ID	020	021	022	023	024	025	026	027	028	029	030	031	032	033	034	035	036	037	038
Demographic data		Age (years)	26	37	31	26	24	42	23	42	27	30	34	43	20	19	22	27	28	24	24
		Sex	Female	Male	Female	Female	Male	Female	Male	Female	Male	Female	Female	Female	Male	Male	Male	Female	Female	Female	Female
	Environmental conditions	Smoking	No	No	No	No	No	No	Yes	No	No	No	No	No	No	No	No	No	No	No	No
		Pet	Yes	No	Yes	Yes	Yes	Yes	Yes	No	Yes	No	No	No	No	Yes	Yes	Yes	Yes	No	Yes
		Family history with AD	No	No	No	AR<br>AC	No	No	FA	AR	AR	Asthma	No	No	No	No	No	AR	AR	AR	Asthma
Allergic rhinitis data (Clinical data)		Age of onset (years)	26	37	31	26	24	5	23	42	27	10	0.58	43	5	3	22	5	10	3	24
		Severity of AR	MI	MoSI	MI	MI	MI	MoSI	MP	MoSP	MI	MI	MoSP	MoSP	MoSP	MoSI	MP	MoSI	MI	MoSP	MoSP
		Current medication	No	No	No	No	No	AH<br>IC	No	AH	No	AH	AH<br>IC	AH	AH	AH	AH<br>IC	AH	AH	AH<br>IC	No
		Comorbidities	No	No	No	No	No	ARC	FA	No	No	No	No	ARC<br>FA	No	No	No	No	No	No	No
	Skin prick test wheal diameter (d), d_{max} x d_{min} (mm)	Bermuda grass (Cd)	0<br>0	0<br>0	0<br>0	0<br>0	0<br>0	4<br>4	0<br>0	2<br>3	4<br>5	0<br>0	0<br>0	3<br>4	3<br>4	3<br>4	3<br>4	4<br>4	3<br>3	0<br>0	3<br>3
		Para grass (Um)	0<br>0	0<br>0	0<br>0	0<br>0	3<br>3	0<br>0	3<br>4	0<br>0	0<br>0	0<br>0	0<br>0	3<br>4	4<br>4	3<br>4	2<br>3	3<br>3	0<br>0	0<br>0	0<br>0
		Johnson grass (Sh)	0<br>0	0<br>0	0<br>0	0<br>0	2<br>3	0<br>0	2<br>2	2<br>2	0<br>0	0<br>0	n.d.<br>n.d.	n.d.<br>n.d.	n.d.<br>n.d.	n.d.<br>n.d.	n.d.<br>n.d.	n.d.<br>n.d.	n.d.<br>n.d.	n.d.<br>n.d.	n.d.<br>n.d.
		Manila grass (Zm)	0<br>0	0<br>0	0<br>0	0<br>0	0<br>0	0<br>0	0<br>0	0<br>0	0<br>0	0<br>0	n.d.<br>n.d.	n.d.<br>n.d.	n.d.<br>n.d.	n.d.<br>n.d.	n.d.<br>n.d.	n.d.<br>n.d.	n.d.<br>n.d.	n.d.<br>n.d.	n.d.<br>n.d.
		Hurricane grass (Bp)	0<br>0	0<br>0	0<br>0	0<br>0	0<br>0	0<br>0	0<br>0	n.d.<br>n.d.	0<br>0	0<br>0	n.d.<br>n.d.	n.d.<br>n.d.	n.d.<br>n.d.	n.d.<br>n.d.	n.d.<br>n.d.	n.d.<br>n.d.	n.d.<br>n.d.	n.d.<br>n.d.	n.d.<br>n.d.
		Sedge (Cm)	0<br>0	0<br>0	0<br>0	2<br>3	3<br>5	3<br>3	3<br>5	2<br>3	2<br>3	4<br>5	3<br>4	3<br>3	4<br>5	0<br>0	3<br>4	3<br>4	3<br>3	3<br>3	0<br>0
		Careless weed (Ah)	0<br>0	0<br>0	0<br>0	3<br>3	2<br>3	0<br>0	2<br>2	3<br>3	0<br>0	0<br>0	4<br>5	2<br>3	3<br>3	3<br>3	3<br>3	3<br>3	0<br>0	0<br>0	0<br>0

Abbreviations: AC, Allergic conjunctivitis; AD/AE, Atopic dermatitis/atopic eczema; ANM, Anosmia; AR, Allergic rhinitis; ARC, Allergic rhinoconjunctivitis; CRR, Chronic/recurrent rhinosinusitis; FA, Food allergy; OSA, Obstructive sleep apnea; MI, Mild intermittent; MoSI, Moderate to severe intermittent; MP, Mild persistent; MoSP, Moderate to severe persistent; AH, Antihistamine; IC, Intranasal corticosteroid; LA, Leukotriene antagonist; IT, Immunotherapy; n.d., No data.

Supplementary Table 1 Demographic and clinical characteristics of allergic rhinitis (AR) patients (n=121). *(cont.)*

		Patient ID	039	041	045	046	047	048	049	050	051	052	053	054	055	056	057	058	059	060	061
Demographic data		Age (years)	24	24	35	37	21	23	47	56	44	24	42	45	57	25	27	59	34	28	27
		Sex	Female	Female	Female	Male	Male	Male	Male	Female	Female	Male	Male	Female	Male	Female	Male	Male	Female	Male	Male
	Environmental conditions	Smoking	No	No	No	No	No	No	No	No	No	No	No	No	No	No	No	No	No	No	Yes
		Pet	Yes	Yes	No	Yes	No	No	No	No	No	No	Yes	No	No	No	Yes	No	Yes	Yes	No
		Family history with AD	AR	AR	AR	No	No	No	Asthma	AR	AR	AR	AC	No	No	No	Asthma<br>AR	No	No	No	No
Allergic rhinitis data (Clinical data)		Age of onset (years)	24	24	15	37	16	1	2	56	10	7	20	2	57	25	27	50	34	28	27
		Severity of AR	MI	MI	MoSI	MP	MoSP	MP	MoSP	MP	MoSP	MoSP	MI	MI	MoSI	MoSI	MoSI	MP	MoSP	MoSP	MoSP
		Current medication	No	No	AH	IC	AH<br>IC	No	AH<br>IC	AH<br>IC	AH	AH<br>IC	AH	AH<br>IC	No	No	AH<br>IC	AH	AH<br>IC	AH<br>IC	AH
		Comorbidities	ARC	No	No	No	No	No	No	No	ARC<br>OSA	ARC	No	No	No	ARC<br>AD/AE	No	No	FA<br>ARC<br>AD/AE<br>CRR<br>OSA	No	No
	Skin prick test wheel diameter (d), d_{max} x d_{min} (mm)	Bermuda grass (Cd)	2<br>3	2<br>2	0<br>0	0<br>0	2<br>3	3<br>4	3<br>3	3<br>4	3<br>4	4<br>4	2<br>2	3<br>3	0<br>0	0<br>0	2<br>3	3<br>3	0<br>0	0<br>0	5<br>7
		Para grass (Um)	2<br>3	3<br>4	3<br>3	3<br>4	3<br>3	3<br>4	2<br>2	2<br>2	0<br>0	4<br>4	2<br>3	0<br>0	3<br>4	0<br>0	2<br>4	3<br>3	3<br>4	3<br>4	0<br>0
		Johnson grass (Sh)	n.d.<br>n.d.	n.d.<br>n.d.	n.d.<br>n.d.	n.d.<br>n.d.	n.d.<br>n.d.	n.d.<br>n.d.	n.d.<br>n.d.	n.d.<br>n.d.	n.d.<br>n.d.	n.d.<br>n.d.	n.d.<br>n.d.	n.d.<br>n.d.	0<br>0	0<br>0	2<br>3	3<br>3	0<br>0	4<br>6	0<br>0
		Manila grass (Zm)	n.d.<br>n.d.	n.d.<br>n.d.	n.d.<br>n.d.	n.d.<br>n.d.	n.d.<br>n.d.	n.d.<br>n.d.	n.d.<br>n.d.	n.d.<br>n.d.	n.d.<br>n.d.	n.d.<br>n.d.	n.d.<br>n.d.	n.d.<br>n.d.	0<br>0	0<br>0	3<br>5	3<br>4	0<br>0	3<br>4	0<br>0
		Hurricane grass (Bp)	n.d.<br>n.d.	n.d.<br>n.d.	n.d.<br>n.d.	n.d.<br>n.d.	n.d.<br>n.d.	n.d.<br>n.d.	n.d.<br>n.d.	n.d.<br>n.d.	n.d.<br>n.d.	n.d.<br>n.d.	n.d.<br>n.d.	n.d.<br>n.d.	0<br>0	0<br>0	2<br>3	0<br>0	0<br>0	2<br>3	0<br>0
		Sedge (Cm)	2<br>2	3<br>3	3<br>3	4<br>5	3<br>3	3<br>4	0<br>0	3<br>3	3<br>3	3<br>3	2<br>2	0<br>0	10<br>11	3<br>4	2<br>3	5<br>6	0<br>0	2<br>3	5<br>6
		Careless weed (Ah)	3<br>3	0<br>0	0<br>0	0<br>0	2<br>2	0<br>0	0<br>0	2<br>3	0<br>0	2<br>2	3<br>3	0<br>0	3<br>3	0<br>0	4<br>5	5<br>6	3<br>5	3<br>4	2<br>2

Abbreviations: AC, Allergic conjunctivitis; AD/AE, Atopic dermatitis/atopic eczema; ANM, Anosmia; AR, Allergic rhinitis; ARC, Allergic rhinoconjunctivitis; CRR, Chronic/recurrent rhinosinusitis; FA, Food allergy; OSA, Obstructive sleep apnea; MI, Mild intermittent; MoSI, Moderate to severe intermittent; MP, Mild persistent; MoSP, Moderate to severe persistent; AH, Antihistamine; IC, Intranasal corticosteroid; LA, Leukotriene antagonist; IT, Immunotherapy; n.d., No data.

Supplementary Table 1 Demographic and clinical characteristics of allergic rhinitis (AR) patients (n=121). *(cont.)*

		Patient ID	062	063	064	065	066	067	068	069	070	072	073	074	075	076	077	078	079	080	081
Demographic data		Age (years)	63	23	22	37	41	41	43	25	25	28	34	24	27	23	44	48	36	37	28
		Sex	Female	Male	Male	Female	Female	Female	Male	Female	Female	Female	Female	Female	Male	Female	Female	Male	Male	Female	Male
	Environmental conditions	Smoking	No	No	No	No	No	No	No	No	No	No	Yes	No	Yes	No	No	No	No	No	No
		Pet	No	No	No	Yes	Yes	Yes	No	No	Yes	Yes	Yes	Yes	No	No	No	Yes	Yes	Yes	Yes
		Family history with AD	No	AR	AR	AR	AR	No	Asthma	No	AR	No	Asthma	AR	No	No	No	No	No	Asthma	No
Allergic rhinitis data (Clinical data)		Age of onset (years)	5	23	1	30	35	41	43	13	25	1	33	1	5	1	8	43	1	6	20
		Severity of AR	MoSP	MoSP	MP	MI	MI	MoSP	MoSP	MI	MoSP	MoSI	MI	MI	MI	MoSI	MI	MoSI	MoSI	MoSP	MP
		Current medication	No	AH<br>IC	AH<br>IC	No	AH	AH<br>IC	AH	AH	No	No	AH<br>IC	AH<br>IC	AH	IC	AH	AH<br>IC	AH<br>IC	AH<br>IC	IC
		Comorbidities	No	No	No	No	No	No	ARC<br>OSA	ARC	FA<br>ARC<br>AD/AE	No	AD/AE<br>Asthma	No	FA	No	No	No	AD/AE	ARC	ARC<br>AD/AE
	Skin prick test wheal diameter (d), d_{max} x d_{min} (mm)	Bermuda grass (Cd)	4<br>4	0<br>0	3<br>4	0<br>0	3<br>4	0<br>0	3<br>4	0<br>0	0<br>0	3<br>3	0<br>0	0<br>0	3<br>4	3<br>3	0<br>0	2<br>2	0<br>0	2<br>4	4<br>4
		Para grass (Um)	4<br>4	0<br>0	3<br>5	0<br>0	0<br>0	0<br>0	4<br>6	0<br>0	0<br>0	0<br>0	4<br>4	0<br>0	3<br>8	5<br>4	0<br>0	2<br>2	2<br>3	2<br>3	3<br>4
		Johnson grass (Sh)	10<br>13	0<br>0	2<br>3	0<br>0	0<br>0	0<br>0	3<br>3	0<br>0	0<br>0	0<br>0	5<br>8	0<br>0	0<br>0	0<br>0	0<br>0	3<br>3	0<br>0	2<br>2	0<br>0
		Manila grass (Zm)	5<br>6	0<br>0	3<br>4	0<br>0	0<br>0	0<br>0	0<br>0	0<br>0	0<br>0	0<br>0	4<br>5	0<br>0	0<br>0	0<br>0	0<br>0	0<br>0	0<br>0	0<br>0	0<br>0
		Hurricane grass (Bp)	10<br>16	0<br>0	0<br>0	0<br>0	0<br>0	0<br>0	5<br>5	0<br>0	0<br>0	0<br>0	4<br>5	0<br>0	0<br>0	0<br>0	0<br>0	0<br>0	0<br>0	0<br>0	0<br>0
		Sedge (Cm)	0<br>0	3<br>4	2<br>3	4<br>5	3<br>3	3<br>4	4<br>5	4<br>5	3<br>5	3<br>4	0<br>0	3<br>3	0<br>0	3<br>3	3<br>4	2<br>2	3<br>4	3<br>5	3<br>4
		Careless weed (Ah)	2<br>4	0<br>0	0<br>0	0<br>0	2<br>2	3<br>4	2<br>3	0<br>0	0<br>0	0<br>0	3<br>3	0<br>0	3<br>4	3<br>3	0<br>0	0<br>0	2<br>2	2<br>3	0<br>0

Abbreviations: AC, Allergic conjunctivitis; AD/AE, Atopic dermatitis/atopic eczema; ANM, Anosmia; AR, Allergic rhinitis; ARC, Allergic rhinoconjunctivitis; CRR, Chronic/recurrent rhinosinusitis; FA, Food allergy; OSA, Obstructive sleep apnea; MI, Mild intermittent; MoSI, Moderate to severe intermittent; MP, Mild persistent; MoSP, Moderate to severe persistent; AH, Antihistamine; IC, Intranasal corticosteroid; LA, Leukotriene antagonist; IT, Immunotherapy; n.d., No data.

Supplementary Table 1 Demographic and clinical characteristics of allergic rhinitis (AR) patients (n=121). *(cont.)*

		Patient ID	082	083	084	085	086	087	088	089	090	091	092	093	094	095	096	097	098	099	100
Demographic data		Age (years)	35	22	20	37	29	19	37	26	36	33	34	35	40	31	18	54	35	27	30
		Sex	Female	Female	Female	Male	Female	Female	Male	Female	Female	Female	Female	Female	Female	Female	Female	Male	Female	Female	Female
	Environmental conditions	Smoking	No	No	No	Yes	Yes	No	Yes	No	No	No	No	No	No	No	No	No	No	No	No
		Pet	No	No	Yes	No	No	Yes	Yes	Yes	No	No	Yes	No	No	No	Yes	Yes	Yes	No	No
		Family history with AD	Asthma<br>AR<br>FA	No	AR	AR	Asthma<br>AR	AR	No	AR	No	No	AR<br>AC	AR<br>FA	AR	No	AR	No	AR<br>FA	No	FA
Allergic rhinitis data (Clinical data)		Age of onset (years)	30	22	13	20	2	0.5	7	20	15	24	20	35	10	0.83	5	53	15	1	1
		Severity of AR	MoSP	MI	MoSI	MoSP	MoSI	MoSP	MoSP	MoSI	MoSI	MoSP	MoSP	MoSP	MoSP	MoSP	MoSP	MP	MI	MI	MI
		Current medication	AH<br>IC	No	AH<br>IC	IC	AH<br>IC	AH<br>IC	AH<br>IC	No	AH<br>IC	AH<br>IC	AH<br>IC	AH	AH<br>IC	AH<br>IC	AH<br>IC	AH<br>IC	AH<br>IC	No	No
		Comorbidities	ARC<br>CRR	No	AD/AE	FA<br>ARC<br>OSA	No	No	No	No	No	AR	ARC	FA<br>ARC<br>AD/AE	ARC<br>AD/AE	No	ARC<br>AD/AE<br>OSA	OSA	AD/AE	No	No
	Skin prick test wheal diameter (d), d_{max} x d_{min} (mm)	Bermuda grass (Cd)	0<br>0	0<br>0	0<br>0	0<br>0	0<br>0	0<br>0	3<br>4	2<br>3	3<br>3	0<br>0	0<br>0	0<br>0	0<br>0	0<br>0	3<br>2	3<br>4	0<br>0	0<br>0	0<br>0
		Para grass (Um)	0<br>0	0<br>0	4<br>5	0<br>0	0<br>0	3<br>3	0<br>0	3<br>3	3<br>4	4<br>4	3<br>4	2<br>2	3<br>5	0<br>0	6<br>5	0<br>0	0<br>0	0<br>0	0<br>0
		Johnson grass (Sh)	0<br>0	0<br>0	0<br>0	0<br>0	0<br>0	0<br>0	0<br>0	0<br>0	2<br>2	4<br>5	0<br>0	0<br>0	4<br>4	0<br>0	0<br>0	0<br>0	2<br>3	0<br>0	0<br>0
		Manila grass (Zm)	0<br>0	0<br>0	0<br>0	0<br>0	0<br>0	2<br>2	0<br>0	2<br>3	0<br>0	3<br>4	0<br>0	0<br>0	2<br>2	0<br>0	2<br>2	0<br>0	0<br>0	0<br>0	0<br>0
		Hurricane grass (Bp)	0<br>0	0<br>0	0<br>0	0<br>0	0<br>0	0<br>0	0<br>0	0<br>0	0<br>0	3<br>4	0<br>0	0<br>0	3<br>3	0<br>0	2<br>2	0<br>0	0<br>0	0<br>0	0<br>0
		Sedge (Cm)	2<br>3	0<br>0	0<br>0	3<br>3	0<br>0	3<br>3	3<br>4	3<br>3	4<br>6	4<br>4	3<br>4	3<br>3	3<br>4	0<br>0	6<br>5	4<br>5	3<br>4	0<br>0	0<br>0
		Careless weed (Ah)	0<br>0	0<br>0	0<br>0	0<br>0	3<br>3	2<br>3	6<br>7	2<br>2	3<br>4	3<br>3	0<br>0	0<br>0	3<br>3	0<br>0	2<br>2	0<br>0	0<br>0	0<br>0	0<br>0

Abbreviations: AC, Allergic conjunctivitis; AD/AE, Atopic dermatitis/atopic eczema; ANM, Anosmia; AR, Allergic rhinitis; ARC, Allergic rhinoconjunctivitis; CRR, Chronic/recurrent rhinosinusitis; FA, Food allergy; OSA, Obstructive sleep apnea; MI, Mild intermittent; MoSI, Moderate to severe intermittent; MP, Mild persistent; MoSP, Moderate to severe persistent; AH, Antihistamine; IC, Intranasal corticosteroid; LA, Leukotriene antagonist; IT, Immunotherapy; n.d., No data.

Supplementary Table 1 Demographic and clinical characteristics of allergic rhinitis (AR) patients (n=121). *(cont.)*

		Patient ID	101	102	103	104	105	106	107	108	109	110	111	112	113	114	115	116	117	118	119
Demographic data		Age (years)	26	56	54	54	37	23	28	29	24	69	61	39	39	40	53	46	29	52	57
		Sex	Female	Female	Male	Female	Female	Male	Female	Male	Female	Female	Female	Female	Female	Male	Female	Male	Female	Female	Female
	Environmental conditions	Smoking	No	No	No	No	No	No	No	No	No	No	No	No	No	No	No	No	No	No	No
		Pet	Yes	No	Yes	No	Yes	No	Yes	No	No	No	No	No	No	No	No	No	Yes	Yes	No
		Family history with AD	AR	AR	AR	Asthma	AR	AR	No	Asthma<br>AR	No	No	AR<br>AC	No	No	No	AR	No	Asthma	No	No
Allergic rhinitis data (Clinical data)		Age of onset (years)	2	55	9	5	3	20	12	15	4	59	1	32	33	32	46	45	26	50	20
		Severity of AR	MoSP	MoSP	MoSP	MP	MoSP	MP	MoSP	MP	MoSP	MoSP	MoSP	MoSP	MoSP	MoSP	MI	MP	MoSP	MoSP	MoSP
		Current medication	AH<br>IC	AH<br>IC	AH	AH<br>IC	No	AH<br>IC	AH	No	AH<br>IC	AH<br>IC	AH<br>IC	AH<br>IC	AH<br>IC	AH<br>IC	IC	No	AH<br>IC	AH<br>IC	AH<br>IC
		Comorbidities	No	OSA	No	ARC<br>AD/AE	ARC	OSA	No	No	No	No	ARC	No	No	OSA	ARC	No	ARC	No	No
	Skin prick test wheal diameter (d), d_{max} x d_{min} (mm)	Bermuda grass (Cd)	0<br>0	0<br>0	7<br>9	3<br>4	0<br>0	2<br>2	8<br>10	3<br>3	3<br>3	0<br>0	4<br>5	3<br>4	0<br>0	0<br>0	0<br>0	0<br>0	0<br>0	0<br>0	7<br>9
		Para grass (Um)	0<br>0	0<br>0	7<br>7	3<br>4	0<br>0	3<br>3	13<br>19	2<br>2	0<br>0	3<br>3	5<br>8	4<br>5	0<br>0	0<br>0	0<br>0	0<br>0	0<br>0	0<br>0	10<br>13
		Johnson grass (Sh)	0<br>0	0<br>0	10<br>15	2<br>3	0<br>0	3<br>4	8<br>9	2<br>3	0<br>0	3<br>4	4<br>6	0<br>0	0<br>0	0<br>0	0<br>0	0<br>0	0<br>0	0<br>0	14<br>24
		Manila grass (Zm)	0<br>0	0<br>0	11<br>17	0<br>0	0<br>0	3<br>4	6<br>7	3<br>4	0<br>0	2<br>3	4<br>5	2<br>3	0<br>0	0<br>0	0<br>0	0<br>0	0<br>0	0<br>0	18<br>16
		Hurricane grass (Bp)	0<br>0	0<br>0	8<br>11	3<br>4	0<br>0	3<br>4	6<br>8	0<br>0	0<br>0	2<br>2	5<br>9	3<br>4	0<br>0	0<br>0	0<br>0	0<br>0	0<br>0	0<br>0	12<br>20
		Sedge (Cm)	4<br>4	0<br>0	7<br>8	3<br>4	4<br>5	3<br>4	4<br>5	3<br>4	3<br>5	0<br>0	3<br>3	4<br>5	0<br>0	0<br>0	0<br>0	0<br>0	0<br>0	0<br>0	0<br>0
		Careless weed (Ah)	0<br>0	0<br>0	8<br>10	0<br>0	3<br>3	3<br>3	2<br>2	3<br>5	2<br>3	3<br>4	5<br>6	0<br>0	0<br>0	0<br>0	0<br>0	0<br>0	0<br>0	0<br>0	4<br>5

Abbreviations: AC, Allergic conjunctivitis; AD/AE, Atopic dermatitis/atopic eczema; ANM, Anosmia; AR, Allergic rhinitis; ARC, Allergic rhinoconjunctivitis; CRR, Chronic/recurrent rhinosinusitis; FA, Food allergy; OSA, Obstructive sleep apnea; MI, Mild intermittent; MoSI, Moderate to severe intermittent; MP, Mild persistent; MoSP, Moderate to severe persistent; AH, Antihistamine; IC, Intranasal corticosteroid; LA, Leukotriene antagonist; IT, Immunotherapy; n.d., No data.

Supplementary Table 1 Demographic and clinical characteristics of allergic rhinitis (AR) patients (n=121). *(cont.)*

		Patient ID	120	121	122	123	124	125	126
Demographic data		Age (years)	48	22	53	33	40	45	61
		Sex	Male	Female	Male	Male	Female	Female	Male
	Environmental conditions	Smoking	Yes	No	No	No	No	No	No
		Pet	No	No	No	Yes	No	No	No
		Family history with AD	No	No	No	No	No	FA	AR
Allergic rhinitis data (Clinical data)		Age of onset (years)	35	18	22	18	35	5	0.67
		Severity of AR	MI	MoSP	MoSP	MoSP	MI	MI	MI
		Current medication	IC	AH<br>IC	AH	AH<br>IC	AH<br>IC	IC	AH<br>IC
		Comorbidities	No	No	OSA	FA<br>Asthma	No	No	No
	Skin prick test wheal diameter (d), d_{max} x d_{min} (mm)	Bermuda grass (Cd)	0<br>0	6<br>8	5<br>6	5<br>7	0<br>0	9<br>12	5<br>5
		Para grass (Um)	0<br>0	8<br>8	4<br>5	8<br>12	0<br>0	8<br>10	0<br>0
		Johnson grass (Sh)	0<br>0	9<br>8	4<br>6	8<br>9	0<br>0	7<br>8	4<br>5
		Manila grass (Zm)	0<br>0	7<br>7	3<br>3	4<br>6	0<br>0	4<br>5	5<br>5
		Hurricane grass (Bp)	0<br>0	8<br>7	3<br>4	5<br>7	0<br>0	6<br>7	5<br>3
		Sedge (Cm)	0<br>0	6<br>5	2<br>2	4<br>5	0<br>0	6<br>7	0<br>0
		Careless weed (Ah)	0<br>0	2<br>2	0<br>0	3<br>3	0<br>0	0<br>0	0<br>0

Abbreviations: AC, Allergic conjunctivitis; AD/AE, Atopic dermatitis/atopic eczema; ANM, Anosmia; AR, Allergic rhinitis; ARC, Allergic rhinoconjunctivitis; CRR, Chronic/recurrent rhinosinusitis; FA, Food allergy; OSA, Obstructive sleep apnea; MI, Mild intermittent; MoSI, Moderate to severe intermittent; MP, Mild persistent; MoSP, Moderate to severe persistent; AH, Antihistamine; IC, Intranasal corticosteroid; LA, Leukotriene antagonist; IT, Immunotherapy; n.d., No data.

Supplementary Table 2 Characteristics of grass and weed pollen species for preparing skin prick test (SPT) extracts.

Type	Grass					Weed	
Family	Poaceae					Cyperaceae	Amaranthaceae
Scientific name	*Cynodon dactylon* (L.) Pers.	*Urochloa mutica* (Forssk.) T.Q.Nguyen	*Sorghum halepense* (L.) Pers.	*Zoysia matrella* (L.) Merr.	*Bothriochloa pertusa* (L.) A.Camus	*Cyperus mitis* Steud.	*Amaranthus hybridus* L.
Common name	Bermuda grass	Para grass	Johnson grass	Manila grass	Hurricane grass	Nutsedge	Careless weed
Inflores-cence	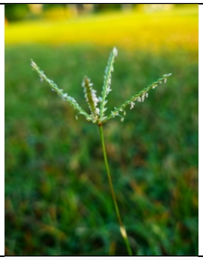	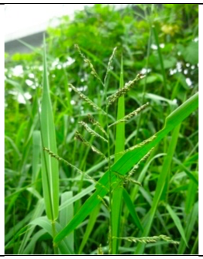	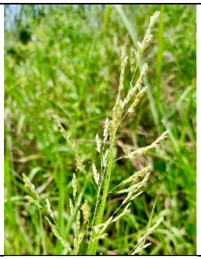	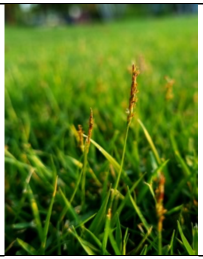	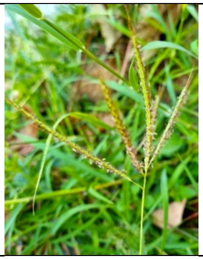	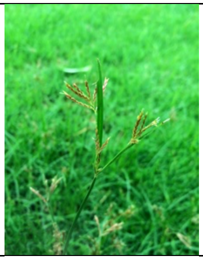	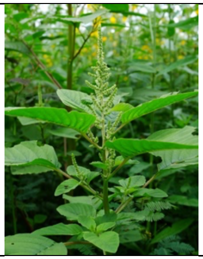
Pollen	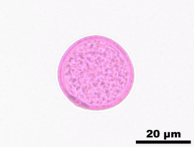	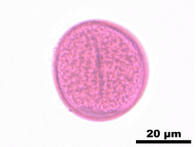	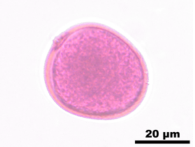	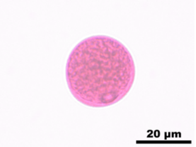	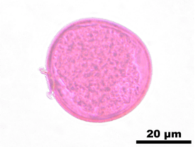	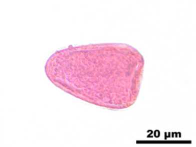	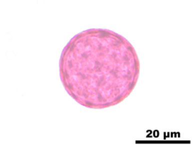
Pollen type	Monad	Monad	Monad	Monad	Monad	Monad	Monad
Pollen shape	Spheroidal	Spheroidal	Spheroidal	Spheroidal	Spheroidal	Wedge	Spheroidal
Aperture	Monoporate	Monoporate	Monoporate	Monoporate	Monoporate	Undefined	Pantoporate
Pollen size, length (µm)†	27.85 ± 1.83	34.48 ± 2.39	38.69 ± 1.00	29.42 ± 2.09	36.35 ± 3.70	34.70 ± 1.02	31.45 ± 1.20
Pollen size, width (µm)†	25.89 ± 1.57	31.84 ± 1.63	34.10 ± 1.45	26.53 ± 2.56	34.41 ± 3.51	25.22 ± 1.71	30.43 ± 1.43
P/E ratio††	-	-	-	-	-	1.38	-

†Pollen size, both width and length, was measured for ten pollen grains at a magnification of 40X (mean ± standard deviation).; ††P/E ratio was calculated using the following equation: pollen polar diameter divided by equatorial diameter.

Supplementary Table 3 Clinical characteristics and age of onset among allergic rhinitis (AR) patients (n=121).

Clinical characteristics	Age of onset	
	< 20 years (n=64)	≥ 20 years (n=57)
**SPT wheal diameter (mm)**		
All species		
Number of patients with +SPT	11	10
Average	2.3	1.6
Median	1.8	1.3
p-value	0.048*	
Bermuda grass (Cd)		
Number of patients with +SPT	35	16
Average	2.5	1.5
Median	3.0	0.0
p-value	0.017*	
Para grass (Um)		
Number of patients with +SPT	34	24
Average	2.9	1.9
Median	3.0	2.0
p-value	0.073	
Johnson grass (Sh)		
Number of patients with +SPT	14	10
Average	2.1	1.3
Median	0.0	0.0
p-value	0.154	
Manila grass (Zm)		
Number of patients with +SPT	12	9
Average	1.7	1.0
Median	0.0	0.0
p-value	0.110	
Hurricane grass (Bp)		
Number of patients with +SPT	13	7
Average	1.8	0.97
Median	0.0	0.0
p-value	0.180	
Nutsedge (Cm)		
Number of patients with +SPT	48	28
Average	3.0	2.4
Median	3.3	2.5
p-value	0.066	
Careless weed (Ah)		
Number of patients with +SPT	22	16
Average	1.7	1.4
Median	1.0	0.0
p-value	0.506	
**Number of species with +SPT**		
Mode	4	4
Median	2	2
**Major type of AR severity**	MoSP	MoSP

p-value was calculated using Mann-Whitney *U* (non-parametric) test.; *p-value is less than 0.05 indicating a statistically significant difference.; +SPT: Positive skin prick test; MoSP: Moderate to severe persistent.

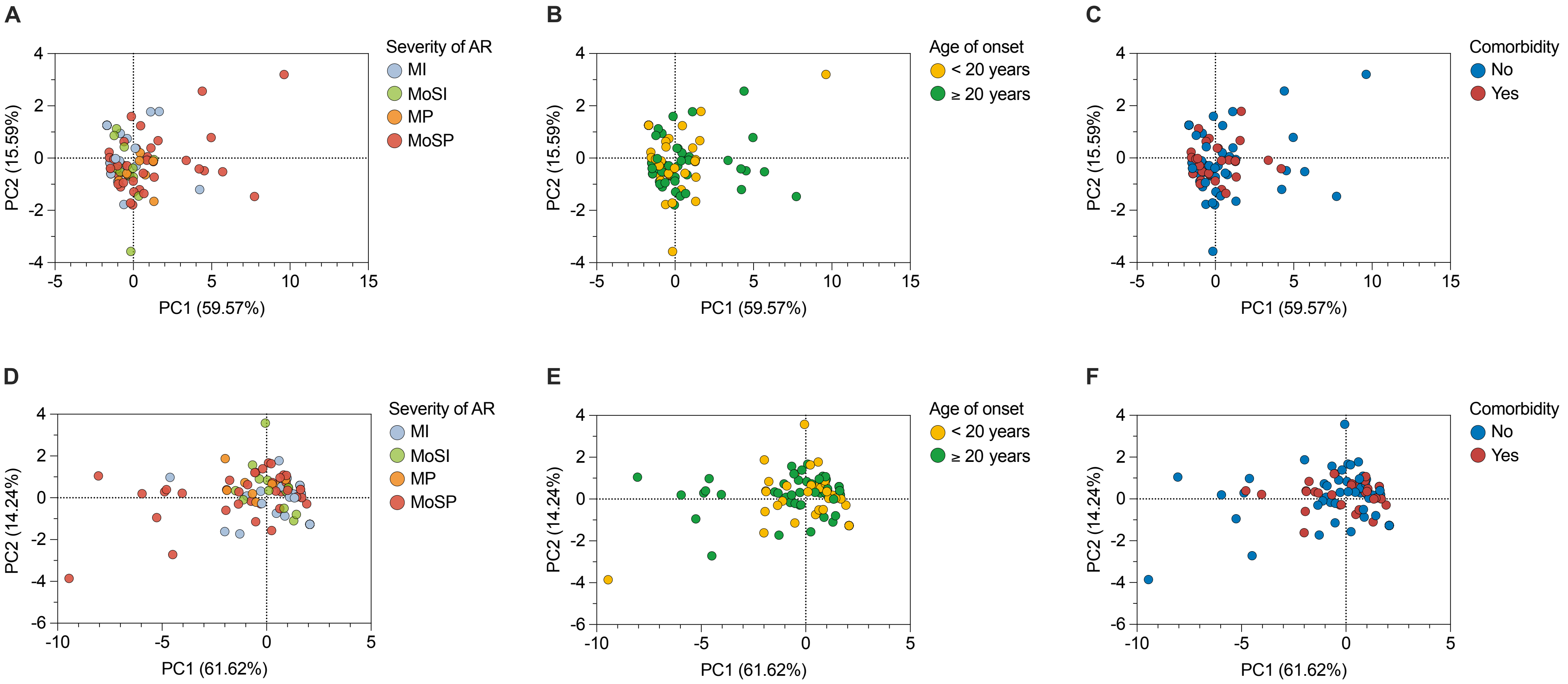

Supplementary Figure 1 Principal component analysis of SPT characteristics for grass and weed pollen. Scatter plots of the two-dimensional principal component analysis (PCA) illustrate the elements of the linear combinations (PC1 score in X-axis and PC2 score in Y-axis) for the **(A-C)** SPT wheal size in response to all grass and weed pollen species, and the **(D-F)** SPT wheal size in response to all grass and weed pollen species and number of species with positive SPT. The coordinates are represented in different colors, indicating the group of **(A and D)** severity of AR: mild intermittent (MI), moderate to severe intermittent (MoSI), mild persistent (MP), and moderate to severe persistent (MoSP), **(B and E)** age of onset: less than 20 years (< 20 years) and more than or equal to 20 years (≥ 20 years), and **(C and F)** comorbidity: without comorbidity (no) and with comorbidity (yes). Of note, Supplementary Figure 1A, and Figure 4A depict identical data sets.
